# Does Coal Mining Have Effects on Land Use Changes in a Coal Resource-Based City? Evidence from Huaibei City on the North China Plain

**DOI:** 10.3390/ijerph182111616

**Published:** 2021-11-04

**Authors:** Jing Guan, Peng Yu

**Affiliations:** 1 School of Geography and Tourism, Anhui Normal University, Wuhu 241002, China; guanjing1010@163.com; 2 School of Tourism and Cuisine, Yangzhou University, Yangzhou 225127, China

**Keywords:** coal mining, high ground water area, land use change, resource-based city

## Abstract

Continuous coal mining results in dramatic regional land use change, and significantly influences the sustainable development of coal resource-based cities. Present studies pay little attention to the characteristics and regularities of land use change in coal resource-based cities, caused by underground coal mining in high groundwater areas. Based on the Landsat remote sensing images of 1999, 2000, 2010, and 2018 of Huaibei City, a typical coal resource-based city of a high ground water area on the North China Plain, this paper applies the dynamic degree and transition matrix of land use to analyze the land use change characteristics, and identify the regularity between land use type and coal mining production in this coal resource-based city. Results show that the land use change in the research area presents an overall characteristic of a constant increase in water area, urban construction land, and rural settlement land, and a continuous decrease in cultivated land. Cultivated land is converted into a water area, urban construction land, and rural settlement land, and rural settlement land and cultivated land are converted bidirectionally. The land use change in this coal resource-based city demonstrates significant reliance on coal resources, and coal mining is significantly related to the area of cultivated land, water area, and rural settlement land, which demonstrates that continuous large-scale coal mining results in damage to cultivated land, a decrease in rural settlement land, and an increase in water area. The research result contributes to the sustainable land use of coal resource-based cities.

## 1. Introduction

Land has always been a crucial resource for human existence and development, and acts as the base and carrier that support human activities [1,2,3]. Land use change is a long-term activity that is based on certain social and economic aims. As an important research area of global change science and sustainable science, it is the most direct representation of terrestrial ecosystem change and the influence of human activities on the earth’s surface system [4,5,6]. The measurement of land use change is a significant part of land resource management and observation. However, most studies often focus on the analysis of land use change on large scales, such as global, region, country, basin, island, or peninsula scales, while less attention is given to land use change on small scales in city or rural areas. Land use change is mainly caused by the complex interaction of human activities and ecological and social factors [7,8]. Present studies mainly probe the influence of macro human activities, such as urbanization and industrialization on land use change [9], while showing less interest in the micro driving force of regional land use change. Due to the difference between different types and degrees of human activities, as well as the heterogeneity of the earth’s surface, land use changes in various areas also present different characteristics and regularities. In this regard, it is important to explore the dynamic change regularities of typical regional land use to promote sustainable regional land use, to further achieve the United Nations (UN) Sustainable Development Goals.

With the fast development of society and the economy, there is a growing demand for coal resources. Large-scale, high strength, and long-term coal mining inescapably affects the regional land use type change [10,11]. A coal resource-based city is one that develops based on exploring, processing, and producing coal resources, and mainly functions as the coal resource supplier. The activities of coal mining are critical driving factors of land use change in coal resource-based cities. However, the differentiation of coal resources determines various coal mining types, including open-cast coal mining and underground coal mining, which further contribute to different regional land use change characteristics and regularities [12,13,14]. The present research pays more attention to the land use change caused by the way of open-cast coal mining, instead of underground coal mining. Furthermore, there are few researchers studying the land use change characteristics in coal resource-based cities caused by coal mining [15,16,17]. Therefore, it is of crucial importance to carry out studies on land use spatial change paradigms in coal resource-based cities, and analyze the relationship between coal mining and land use type, further disclosing the interaction mechanism of regional land use change and coal mining.

However, there are still some limitations in the relative studies on land use change in coal resource-based cities. First, present studies tend to probe the transformation of the regional terrestrial ecological environment to a water ecological environment caused by underground coal mining in specific coal areas, instead of analyzing the land use change resulting from coal mining in populated, developed, high ground water areas on the plain from the perspective of the city. Second, land subsidence caused by coal mining is a long-term and complex evolution. Present studies fail to present the new characteristics of land use change after the transformation development in coal resource-based cities in today’s era, which is facing structural transformation from energy resource use, obstacles of ecological environment, and constraints of traditional industry development. Therefore, the relevant remote sensing image data must also be updated. Lastly, the externality of coal mining greatly affects the land use change in coal resource-based cities. Moreover, the correlation analysis of land use type change and total coal production in coal resource-based cities is still unclear, and the relationship needs to be further illustrated.

Compared with the energy structures of other developed countries, the resource characteristics—coal-rich, oil- and gas-poor—contributes to China’s long-term energy structure, which prioritizes coal. China is the largest coal-consuming country in the world, and approximately 90% of its total coal production comes from underground coal mining. Mining subsidence is the serious problem in the mining process [18]. There are great compositive areas of underground coal resources, cultivated land, and urban construction land and rural settlement land in high ground water areas of eastern China [19]. The subsidence caused by accumulated underground coal mining results in large-scale water-logged and inundated land in coal areas of the China Plains, which further leads to the decline in cultivated land and sharp conflicts between population, land, and agriculture [16]. In this regard, this paper selects Huaibei City—a coal resource-based city on the North China Plain—as an example to explore the characteristics and influencing factors of land use change, and attempts to answer the following questions: (1) is there any regularity in the mutual transformation of land use types in the coal resource-based city in high ground water areas on the plain with long-term scale? (2) What is the relationship between the amount of coal mining and land use types?

## 2. Research Method and Data Collection

### 2.1. Background of Research Area

**Figure 1:**
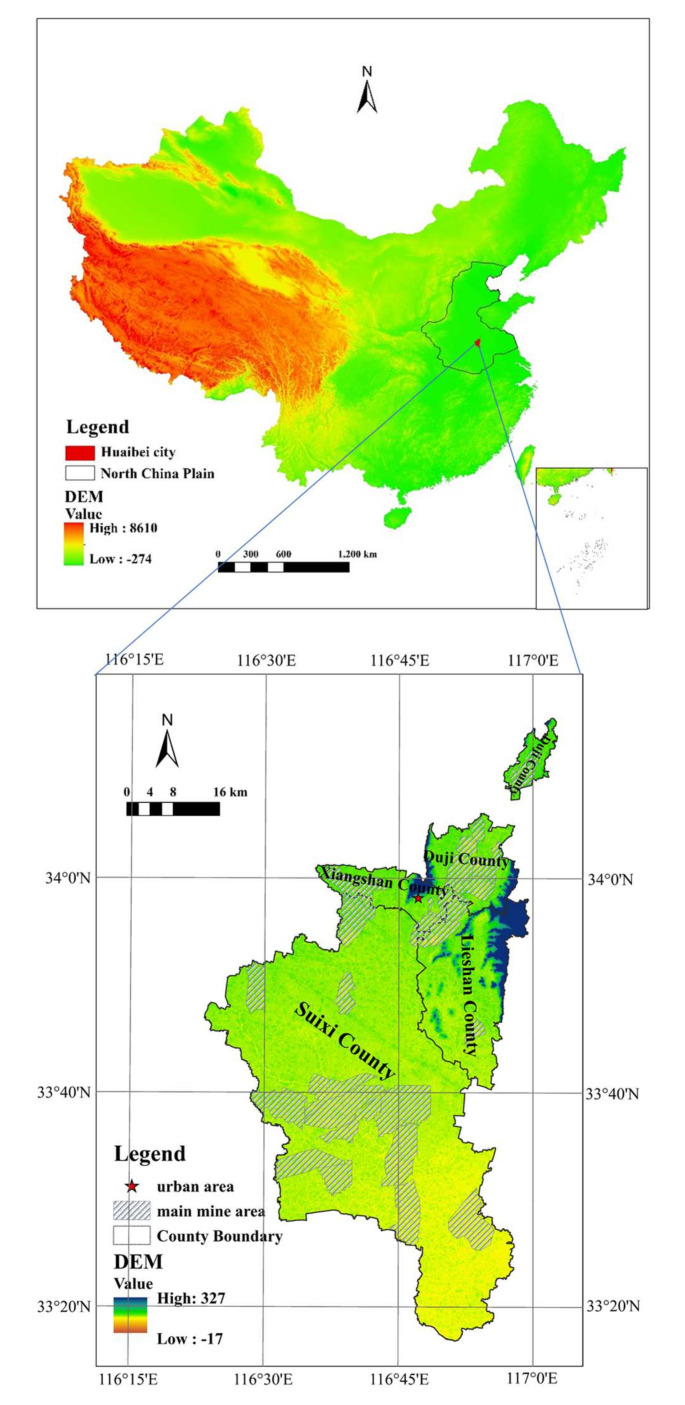
Huaibei’s geographical location in China (note: the figure is drawn by the author).

The North China Plain is low and flat, with the majority of its land under 50 m above sea level, and is the most populous plain, making up approximately 24.2% of the total population in China. It is also the major grain-producing area, and an important high ground water area in China. As a typical mine–grain mixed zone, the North China Plain produces both coal and grain [20]. Huaibei City, a city of Anhui Province, is located in the North China Plain (Figure 1).

**Figure 2:**
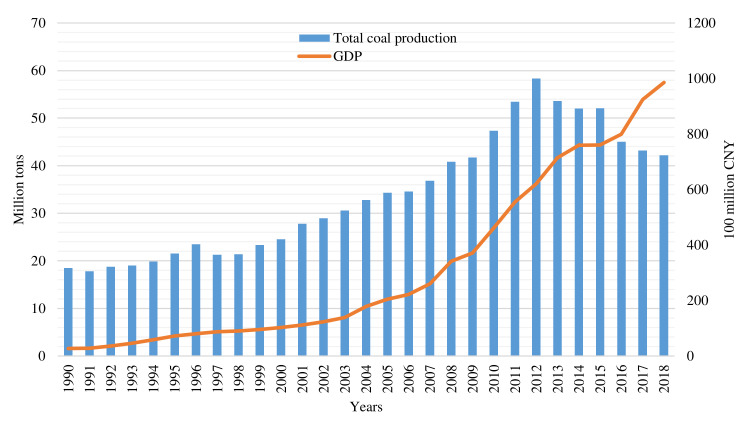
The total coal production and GDP in Huaibei City from 1990 to 2018.

Huaibei City, with a total land area of 2741 km^2^, was founded in 1957 for coal mining. It is a typical coal resource-based city that governs three districts and one county, namely Xiangshan district, Duji district, Lieshan district, and Suixi county. It is a city with a dense population and a well-developed economy, whose permanent population density is 792 per square kilometers; the permanent population urbanization rate reached 65.9%, and the industrialization rate was 39% in 2019. At present, there are 23 large coal mines in Huaibei City, with an average annual total coal production of 33.97 million tons from 1990 to 2018 (Figure 2). Moreover, the largest annual coal production in the city occurred in 2012; since then, coal production has fallen markedly. On the one hand, China’s economic growth has slowed since 2012, which has led to a decline in the demand for coal production. On the other hand, Huaibei City was listed as a resource-exhausted city by the state in 2009, and the amount of coal to be mined was insufficient (approximately 80% of the mines are in the coal-resource depletion phase). Under the influence of these two factors, the annual coal production in Huaibei City declined after 2012, and this trend will exist for a certain period of time.

**Figure 3:**
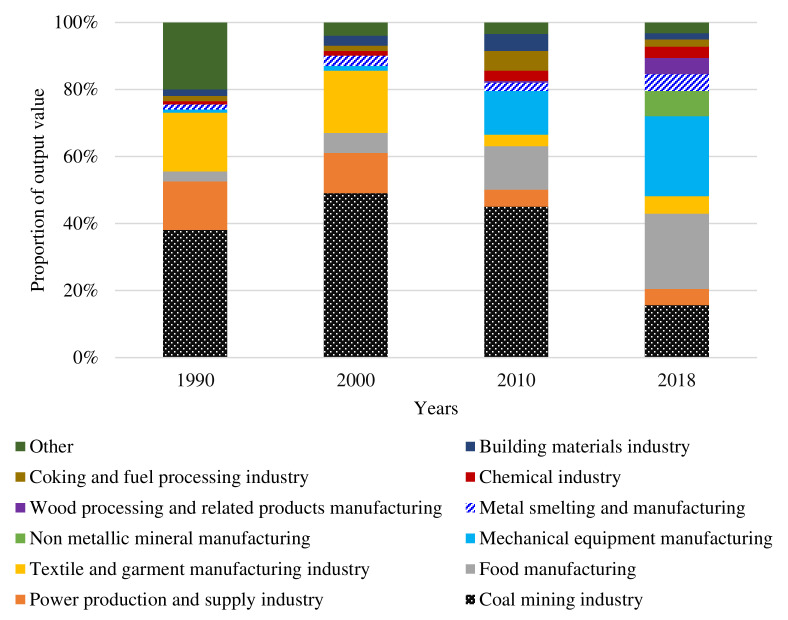
Structure of output value of major industrial sectors in Huaibei City.

Huaibei City’s underground water level is 2–3 m, the annual average temperature is 14.5 °C, and the annual average amount of precipitation is 862.9 mm. As a coal–grain mixed zone in high ground water areas, land use in Huaibei prioritizes agriculture before coal mining development. With the development of coal mining, the coal industry has gradually become the pillar industry in the economic development of Huaibei since the 1990s. After 2010, the city entered a transition period, and the proportion of coal industry output value gradually decreased (Figure 3). With constant large-scale coal mining, the cultivated land is in the process of dynamic subsidence, which further results in waterlogging and becomes a water body, increasing the proportion of affected cultivated land. In the future, coal resource mining activities will continue, and their impact on land use will continue to exist.

### 2.2. Research Method

The data analysis methods in this article include land use dynamic degree (in order to describe the rate of change of a certain land use type in a region) and land use intensity (in order to describe the degree of change of a certain land use type during the study period). The specific formulas are as follows:

(1) $$K=\left[\frac{U_{b}-U_{a}}{U_{a}}\right]\times \frac{1}{T}\times 100\%$$

where K indicates the dynamic degree of a certain land use type during the study period, U_a_ indicates the area of a certain land use type at the beginning of the study period, U_b_ indicates the area of a certain land use type at the end of the study period, and T indicates the length of study period.

(2) $$S_{i}=\frac{U_{i}}{\left(U\times T\right)}$$

where S_i_ indicates the land use intensity of a certain land use type during the study period, U_i_ indicates the absolute change of the i-land type within the study period, U indicates the absolute change of all land types in the study area during the study period, and T indicates the length of study period.

### 2.3. Data Collection

The research data are mainly from the Landsat remote sensing image of Huaibei City in 1990, 2000, 2010, and 2018, with a spatial resolution of 30 × 30 m. The choice of research period mainly considers the availability of data, and the phased characteristics of Huaibei City’s development. Although annual coal production began declining in 2012, the city has entered a transitional stage of development since 2010. Therefore, it is reasonable to select the year 2010 in this research. This paper applies the methods of geometric correction and image enhancement to process the remote sensing image, and adopts the human–machine interactive visual interpretation to extract the land use data of Huaibei. Referring to the classification of the International Geosphere–Biosphere Programme (IGBP), this paper divides the land use types in Huaibei City into six types, including cultivated land, forest land, grass land, water area, urban construction land, and rural settlement land, based on the actual situation of the case. In addition, the socio-economic data of this paper are primarily from the Huaibei City Statistical Yearbook [21].

## 3. Results

### 3.1. Stage Division of Land Use Change

**Figure 4:**
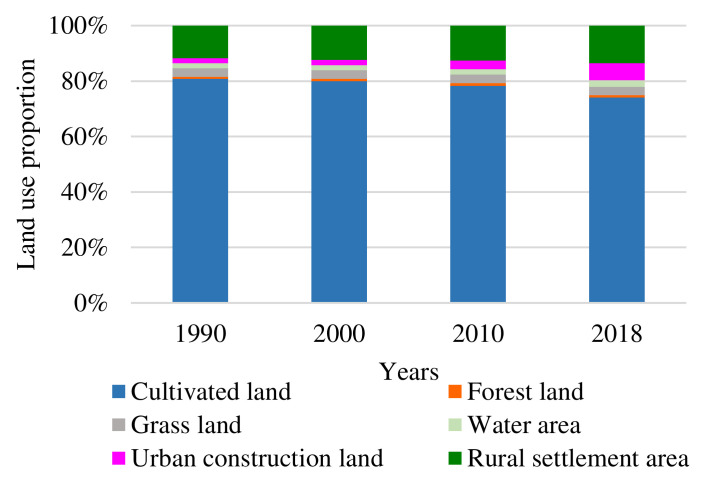
The total coal production and land use type in Huaibei City.

Based on the analysis of total coal production, different land use type change and socio-economic development background in research area over the years (Figure 4), this research proposes that the land use change is generally divided into three stages, including low-speed dispersion development stage, medium-speed aggregation development stage, and high-speed equilibrium development stage. (1) During the low-speed dispersion development stage (from 1990 to 2000), the speed of land use change was low, and the development in coal resource-based cities mainly focused on the coal mining industry. Under the mainly influence of China’s planned economic system, Huaibei City had a single industrial structure (Figure 3), and the extent of the city’s economic development was low. The mining scope of this stage is small, and the influence of mining activities on the overall pattern of land use is not significant. (2) In the medium-speed aggregation development stage (from 2000 to 2010), the overall speed of land use change was faster. In the context of China’s market economic system, the development of the coal industry and non-coal industry is accelerating in the city. Coal production increased from 24.59 million tons in 2000 to 47.35 million tons in 2010, and industrial output increased from CNY 3.84 billion in 2000 to CNY 27.37 billion [21]. The development of the industry has led to the expansion of the city’s economic scope and population concentration. The urbanization process in Huaibei has accelerated, with the number of urban residents increasing from 0.73 million in 2000 to 1.161 million in 2010, which further promoted the rapid transformation of land types, such as cultivated land into urban construction land. (3) During the high-speed equilibrium development stage (from 2010 to 2018), the speed of land use change accelerated significantly. The total coal production first increased and then decreased, coal-related industries gradually declined, and the effect of coal resources on land use change was also weakened due to coal resource reserve shortages. However, under the influence of the central government support policy (by 2018, Huaibei City has received a total of approximately CNY 5.4 billion in financial transfer funds from the state), the city has carried out transformation and upgrading, the speed of urban construction has accelerated, and changes of the various types of land use in the city have been drastic.

### 3.2. Rate of Change of Different Land Use Types

According to dynamic degree and change intensity index models, this paper calculates the dynamic degree and change intensity index of each type of land use in all the stages in Huaibei City (Table 1). The results demonstrate that the overall land use structure in the city has dramatically changed since 1990, especially the decrease in cultivated land, and the increase in urban construction land and rural settlement land and water area.

The leading role of cultivated land was not significantly changed, although its area plummeted to 177.82 km^2^ from 1990 to 2018. The changing intensity in different stages was significantly higher than other land use types. The dynamic degree of cultivated land improved from −0.07% at the low-speed dispersion development stage to −0.61% at the medium-speed aggregation development stage, and presented a constant accelerating decrease and decreasing pace characteristic. On the other hand, the urban construction land and rural settlement land continuously increased and showed an acceleration tendency, especially at the high-speed equilibrium development stage with a significant expansion area of 108.65 km^2^—this was also observed for water land. Due to the promotion of coal mining development in Huaibei City, the changing intensity of water area has been continuously increasing since 2000. Both forest land and grass land presented the tendency of first increasing, and then declining, even though they were from comparatively minor bases.

### 3.3. Dynamic Conversion between Different Land Use Types

**Figure 5:**
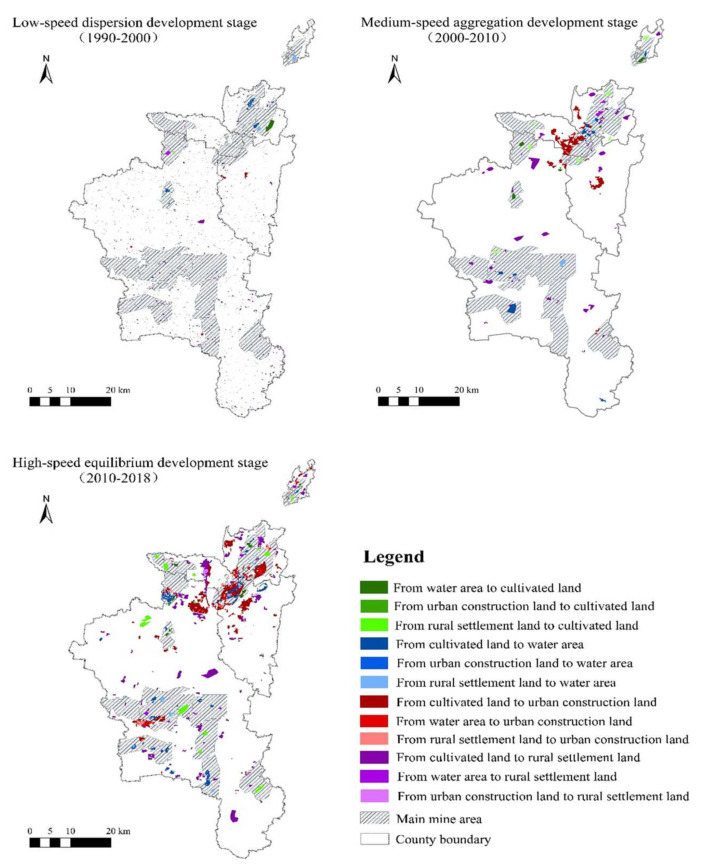
Land use type conversion spatial distribution map of Huaibei City (note: the figure is drawn by the author).

To better reflect the land use types conversion, this paper probes the transition matrix (Table 2), and spatial distribution of land use change at different stages (Figure 5). Moreover, the table and figure only list the land use types that go through great change.

Based on the data analysis, this paper concludes that coal mining results in an increase in water land, which is mainly converted from cultivated land and rural settlement land. There was approximately 6.90 km^2^, 9.57 km^2^, and 28.86 km^2^ of cultivated land and rural settlement land being converted into water area at the low-speed dispersion development stage, medium-speed aggregation development stage, and high-speed equilibrium development stage, respectively. This paper illustrates the stacking chart of water area change and major mine distribution to explore the relationship between the increase in water area and coal mining, and concludes that the distribution of increased water area is basically in line with major mine distribution, which further discloses that the increase in water area is closely related to the coal mining.

In addition, the increase in urban construction land and rural settlement land comes at the expense of a decrease in cultivated land. It is estimated that almost 199.04 km^2^ of cultivated land was converted into construction and settlement land in the past 29 years, despite the fact that there were 15.00 km^2^, 13.29 km^2^, and 28.17 km^2^ of other land use types being converted into cultivated land at the low-speed dispersion development stage, medium-speed aggregation development stage, and high-speed equilibrium development stage, respectively, which could have largely contributed to the land reclamation in the coal resource-based city. Huaibei City has implemented land reclamation since 1985, and has initiated many reclamation patterns such as deep digging and filling shallow

### 3.4. The Effect of Coal Mining on Land Use Types Change

**Figure 6:**
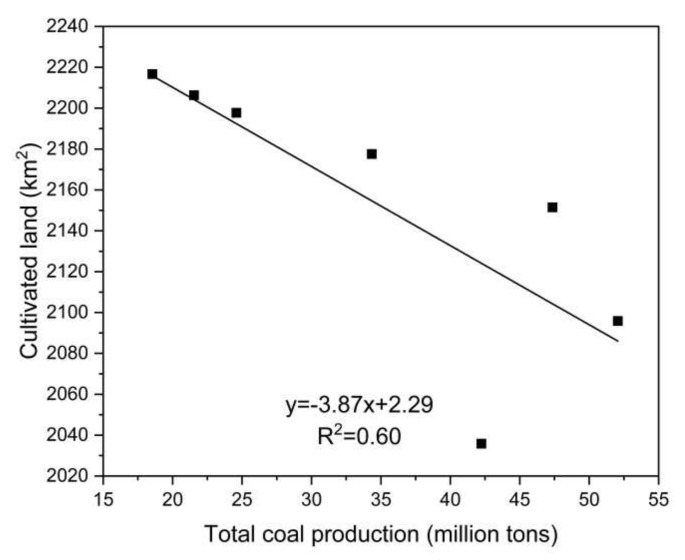
The relationship between total coal production and cultivated land.

**Figure 7:**
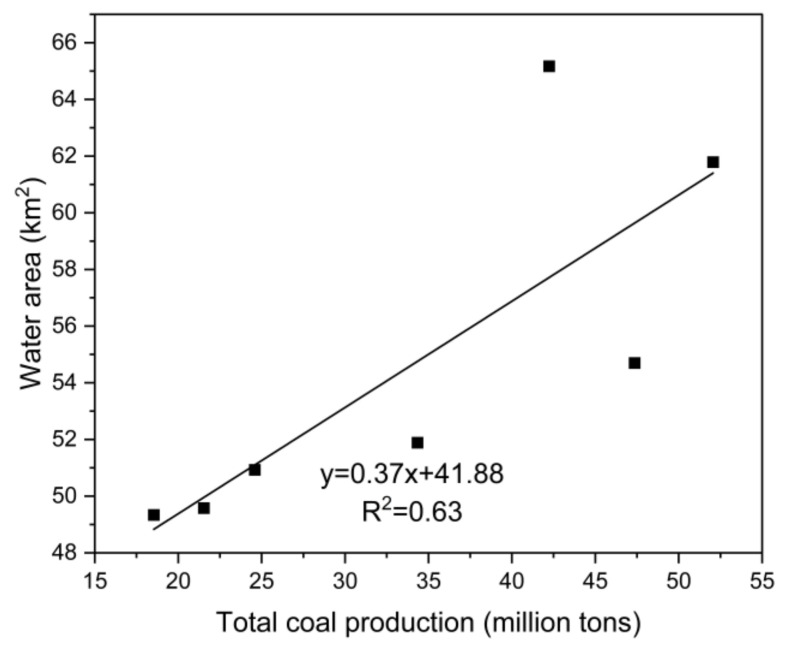
The relationship between total coal production and water land.

**Figure 8:**
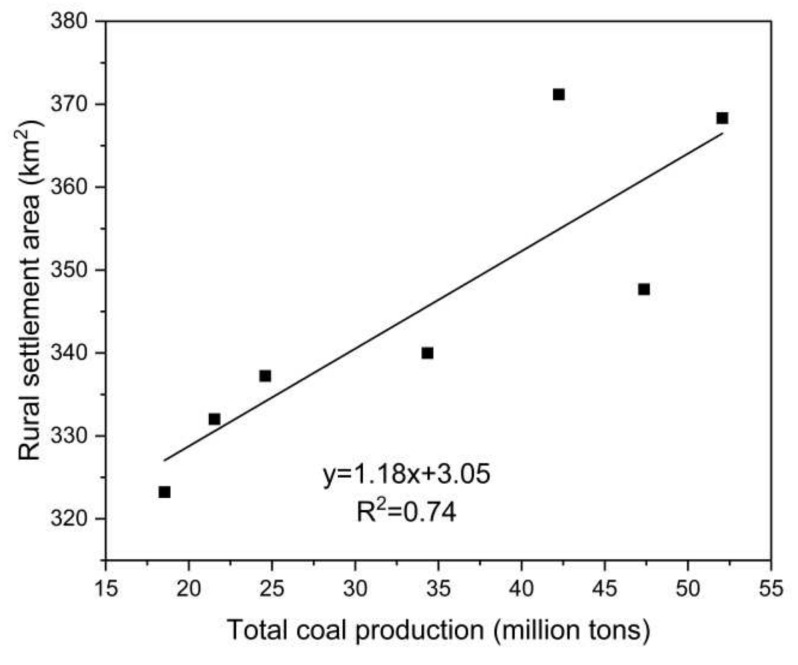
The relationship between total coal production and rural settlement land.

Coal mining is the most prominent driving force of land use change in resource-based cities. Different from other city types, the development of coal resource-based cities presents significant resource-related reliance and orientation, and coal mining causes changes to the region of different land use types. Therefore, this paper analyzes the relationship between total coal production and cultivated land, forest land, grass land, water area, urban construction land, and rural settlement land in 1990, 1995, 2000, 2005, 2010, 2015, and 2018, respectively. This paper concludes that cultivated land, water area, and rural settlement land correlate significantly with total coal production (Figure 6, Figure 7 and Figure 8). There is a significant positive correlation between water area and total coal production (*r* = 0.791, *p* = 0.034), rural settlement land, and total coal production (*r* = 0.863, *p* = 0.012), and a significant negative correlation between cultivated land and total coal production (*r* = −0.773, *p* = 0.042). However, there is no significant correlation between urban construction land and total coal production (*r* = 0.712, *p* = 0.072), forest land and total coal production (*r* = 0.744, *p* = 0.055), and grass land and total coal production (*r* = −0.673, *p* = 0.097). These results demonstrate that constant coal mining and the accumulation of total coal production contribute to the continuous expansion of water area and rural settlement land, as well as a constant decrease in cultivated land in the research area.

Subsidence is the most significant externality caused by underground coal mining in high ground water areas on a plain. Constant underground coal mining and eventual mining result in the failing of ground support, leading to surface land subsidence. Due to the difference between deep subsidence and land nature, subsidence causes different land changes from seasonal and perennial waterlogging to subtle land deformation. Seasonal and perennial water logging converts cultivated land, forest land, and construction or settlement land into wetland, which completely changes the land use type and increases the area of water area in the research area directly. Mining subsidence further influences the research area—water area, rural settlement land, and cultivated land. In this regard, the former three land use types are significantly correlated with coal mining. There is no significant correlation between urban construction land and coal mining, due to the following two aspects. First, the construction of urban construction land is generally based on the planning of the government, instead of the layout of a coal mine. Second, urban land use change is a result of multiple factors such as urbanization and government behavior, rather than the single effect of the coal mining industry. Furthermore, forest land and grass land are not significantly correlated with total coal production, mainly due to the scarcity of research area of these two land use types, thus there is no stable change regularity.

## 4. Discussion and Conclusion

### 4.1. Discussion

#### 4.1.1. Land Use Changes in Coal Resource-Based Cities

Underground coal mining exerts a far-reaching influence on land use change in coal resource-based cities in high ground water areas on a plain. China’s energy structure, dominated by coal, will not change in the short term. Moreover, the influence of coal mining on land use continues in many other coal-producing countries [22]. There are three major aspects of land use change that are affected by coal mining in high ground water areas of coal-producing countries.

The first is the worsening decline of cultivated land. Coal mining not only causes the subsidence of cultivated land and the loss of soil nutrients, which directly causes the reduction of cultivated land area and the decrease of soil quality in the research area, but also increases the content of heavy metal pollutants in water sources, which leads to the reduction of crop yield. The destruction of high-quality cultivated land caused by coal mining threatens regional, national, and even global, food security [23,24]. There were more than 0.268 million hectares of high-quality cultivated land damaged due to coal mining in the research area by the year 2018, with an urban per capita cultivated land area of 0.062 hectares, slightly above the minimum size of 0.053 hectares recommended by the United Nations Food and Agriculture Organization (UN-FAO).

The second is the expansion of water area with the development of coal mining. The formation of subsidence water land and its expansion alters the city water structure, and influences the city’s ecosystem [25]. The terrain in the research area was previously dominated by plains and had few landscape types, such as wetland and lakes, before coal mining. However, the large-scale subsidence of water land—resulting from coal mining—was converted into a wetland landscape with collapsed lakes, which increased the city’s water area.

The third is the destruction of rural settlement land. Mining subsidence forces a large number of villagers to move out. Since 1990, there have been 275 villages moving due to mining subsidence—more than 0.3 million people involved in the research area. Village moving in coal mining areas reconstructs the living space and converts the lifestyle of rural residents [26]. This new movement pattern leads to increased construction land that is smaller than the released land after moving, which contributes to the rural settlement land conservation, mitigates rural-urban land use conflict, and optimizes the urban land use structure. In 2009–2020, the relocation of villages in coal mining collapse areas could save 1275.55 hm^2^ of land in the research area.

In addition, this paper discloses that there is no significant correlation between coal mining and urban construction land change, which is different from previous scholars, who hold that urban construction land in resource-based cities is significantly correlated with coal mining [27,28]. This is mainly due to the co-effectiveness of economic development and government regulation on urban construction land in coal resource-based cities in China. Influenced by the city’s development planning, as well as the fiscal reliance on land, local government activates the real estate industry (real estate investment in Huaibei increased nearly 50-fold from 264 million in 2000 to 13.169 billion in 2018), which results in uncontrolled expansion and increased speed of construction land in the city [29]. This is highlighted in the fast expansion of urban construction land, even though the coal production decreased and economic growth slowed down (Huaibei’s economic growth rate fell to 6.62% from 24.14%) in the city’s transition development period.

#### 4.1.2. Impact on Coal Resource-Based Cities Development Planning

The complex land use change in coal resource-based cities exerts a profound influence on the social economy, such as construction land destruction, cultivated land degeneration, and moving villages in coal mining areas. Coal resource-based cities need to coordinate the conflicts between mining, land reclamation, cultivated land protection, and city development before land exploitation and land use.

First, a plan for reclamation in advance and safeguard food security should be constructed. Despite the results that the present cultivated land protection policy has achieved, the degeneration of high-quality farmland caused by mining subsidence greatly threatens local food security [30]. Accelerating land reclamation is a comparatively effective way to recover the losses. Land reclamation in collapsed areas is of great practical significance for restoring cultivated land, repairing the ecological environment, and alleviating land use contradictions. Land reclamation in China was developed in practice in the early 1980s. The state has made compulsory provisions for land reclamation and ecological restoration in abandoned industrial and mining areas, and has promulgated relevant laws and regulations such as the Provisions on Land Reclamation (1988) and the Regulations on Land Reclamation (2011). However, traditional land reclamation carried out after land subsidence is lengthy, and has comparatively low efficiency. In this regard, it is important to consider production and land reclamation, integrate mining and reclamation, and develop overall planning of mining and reclamation, to achieve the goal of cultivated land protection before complete subsidence, reduce the reclamation time of disturbed land, and promote the land reclamation efficiency.

Second, reasonable city planning can improve land use efficiency. China is in the stage of rapid urbanization, and the conflicts between development, coal mining, ecological optimization, and cultivated land protection need to be highlighted in city planning. It is of crucial importance to predict the land use type after coal mining accurately, and grasp the rules of land use to formulate city planning in coal resource-based cities [31]. According to the statistics, more than one-third of underground land is coal-bearing land in the research area. The distribution of villages is generally in line with the distribution of mines, which results in the destruction of local residents’ living environment. Moreover, forced evacuation causes land resource waste. Therefore, it is significant to develop coordinated planning of land reclamation and village moving in coal mining areas, and optimize the regional land use efficiency.

Third, water areas should be used rationally, and the ecological environment optimized. Mining subsidence enables single terrestrial landscapes to transform into terrestrial–wetland landscapes, which enhances the diversity of land use type, and enhances the anti-jamming capacity of the ecosystem [32]. In this regard, formulating reasonable planning and utilizing subsidence water land contributes to the city’s ecological environment protection. In addition, the government should integrate ecological management concepts into all parts of land planning, including design, formulation, implementation, and feedback.

### 4.2. Limitations and Recommendations for Further Study

This paper has achieved certain progress in exploring the influence of coal mining in high ground water areas on land use change in a coal resource-based city, while failing to undertake further research on the following three topics. First, this study does not establish separate classification standards of land use type for coal mining and other uses, which might help to accurately measure the influence of coal mining on land use. Second, the present study does not probe the influence of land use change on ecosystem service in a coal resource-based city [33,34]. Third, this paper does not explore the coupling relationship between land use change and social economy in a coal resource-based city, especially the influence of coal mining on residents’ livelihood transitions. It is also worth considering the costs related to industrial activity—mining damages and reclamation versus profits related to mining in future research.

### 4.3. Conclusions

Based on the adoption of RS and GIS, this paper takes Huaibei City on the North China Plain as a case to analyze the influence of coal mining in a high ground water area on land use change in a coal resource-based city, and examines the relationship between coal mining production and the dynamic change of land use type in Huaibei City, which provides support for decisions of regional sustainable land use and socio-economic development.

The significant expansion of urban construction land and water area, and constant decrease in cultivated land are major characteristics of land use change in coal resource-based cities. There are frequent conversions between different land use types, mainly dominated by cultivated land converted into construction and settlement land. Compared with other type cities, land use structure change is faster and more complex in coal resource-based cities, due to mining subsidence. The contradiction between land supply and demand becomes even more acute, and severely threatens the sustainable development of cities.

There is an apparent periodical characteristic in the land use of coal resource-based cities. The land use change is generally divided into three stages, including (1) the low-speed dispersion development stage, (2) the medium-speed aggregation development stage, and (3) the high-speed equilibrium development stage. The land use change in coal resource-based cities demonstrates significant reliance on resources, and coal mining is significantly related to the area of cultivated land, water area, and rural settlement land, which demonstrates that continuous large-scale coal mining results in damage to cultivated land, decrease in rural settlement land, and increase in water area.

## Figures and Tables

**Table 1:** Land use change rate and land use intensity at different stages.

Land Use Type	Low-Speed Dispersion Development Stage (1990–2000)			Medium-Speed Aggregation Development Stage (2000–2010)			High-Speed Equilibrium Development Stage (2010–2018)		
	Variation (km^2^)	Land Use Dynamic Degree (%)	Land Use Intensity (%)	Variation (km^2^)	Land Use Dynamic Degree (%)	Land Use Intensity (%)	Variation (km^2^)	Land Use Dynamic Degree (%)	Land Use Intensity (%)
Cultivated land	−18.95	−0.07	4.94	−46.24	−0.20	4.99	−115.63	−0.61	6.05
Forest land	0.77	0.38	0.20	2.02	0.95	0.22	−1.51	−0.65	0.08
Grass land	0.07	0.01	0.02	0.01	-	0.00	−2.52	−0.29	0.13
Water area	1.60	0.38	0.41	3.77	0.7	0.42	10.47	2.14	0.55
Urban construction land	3.02	0.62	0.79	30.12	5.83	3.25	85.16	10.41	4.46
Rural settlement land	13.98	0.44	3.64	10.45	0.34	1.13	23.49	0.68	1.23

**Table 2:** Land use transition matrix at different stages.

Research Period	Land Use Type	Cultivated Land (km^2^)	Water Area (km^2^)	Urban Construction Land (km^2^)	Rural Settlement Land (km^2^)
Low-speed dispersion development stage (1990–2000)	Cultivated land	2179.34	5.86	2.74	24.35
	Water area	4.22	44.97	0.04	1.08
	Urban construction land	0.38	0.04	48.17	0.01
	Rural settlement land	10.40	1.04	0.02	312.28
Medium-speed aggregation development stage (2000–2010)	Cultivated land	2153.52	6.57	28.97	21.99
	Water area	3.14	45.75	0.65	2.01
	Urban construction land	0.03	0.00	51.64	0.01
	Rural settlement land	10.12	3.00	0.43	336.68
High-speed equilibrium development stage (2010–2018)	Cultivated land	2020.84	22.81	62.61	58.38
	Water area	6.32	35.73	8.35	4.46
	Urban construction land	0.21	0.01	79.53	2.04
	Rural settlement land	21.64	6.05	9.24	332.80

## Data Availability

Not applicable.
